# Re‐salvage focal low‐dose rate brachytherapy for local recurrence of prostate cancer after salvage focal low‐dose rate brachytherapy

**DOI:** 10.1002/iju5.12670

**Published:** 2023-11-22

**Authors:** Takahiro Nakamoto, Takashi Yoshida, Toshiko Shiga, Makoto Taguchi, Takao Mishima, Shigenari Kawakita, Takashi Murota, Hidefumi Kinoshita

**Affiliations:** ^1^ Department of Urology and Andrology Kansai Medical University Osaka Japan; ^2^ Department of Radiology Kansai Medical University, Medical Center Osaka Japan; ^3^ Department of Urology and Andrology Kansai Medical University, Medical Center Osaka Japan

**Keywords:** brachytherapy, local recurrence, salvage therapy

## Abstract

**Introduction:**

Salvage brachytherapy represents an effective treatment for local recurrence of prostate cancer after prior external beam radiotherapy. However, the optimal therapeutic strategies for local recurrence after salvage brachytherapy have not yet been determined.

**Case presentation:**

We describe the case of a 77‐year‐old man who underwent re‐salvage focal low‐dose rate brachytherapy for local recurrence after carbon ion radiotherapy and salvage focal low‐dose rate brachytherapy. We performed re‐salvage focal low‐dose rate brachytherapy for the recurrence with a different type of seed, which resulted in a significant reduction in the prostate‐specific antigen level. During the 35‐month follow‐up after re‐salvage focal low‐dose rate brachytherapy, no recurrence of prostate cancer and no severe radiation‐related toxicities were observed.

**Conclusion:**

Our patient was successfully treated with re‐salvage focal low‐dose rate brachytherapy for local recurrence after salvage focal low‐dose rate brachytherapy. This treatment strategy might be effective for such patients and is not associated with sexual dysfunction or severe adverse events.

**Abbreviations & Acronyms**
ADT: androgen deprivation therapy
BCR: biochemical recurrence
CT: computed tomography
*D*_90_
: dose delivered to 90% of the PTV
fLDR: focal low‐dose rate
GI: gastrointestinal
GS: Gleason score
GU: genitourinary
HDR: high‐dose rate
LDR: low‐dose rate
LR: local recurrence
MRI: magnetic resonance imaging
PCa: prostate cancer
PSA: prostate‐specific antigen
PSADT: prostate‐specific antigen doubling time
PSMA‐PET: prostate‐specific membrane antigen positron emission tomography
PTV: planning target volume
*R*_100_
: rectal volume received 100% of the prescribed dose
UD_30_
: dose: received by 30% of the urethra
*V*_100_
: volume of PTV: receiving 100% of the dose

**Keynote message**

The optimal therapeutic strategies for locally recurrent prostate cancer after focal salvage brachytherapy have not been determined. Re‐salvage focal low dose brachytherapy might be one of beneficial options for local recurrence after salvage focal low dose brachytherapy with favorable oncological outcomes and preservation of sexual function.

## Introduction

Among patients who undergo radical radiotherapy for PCa, 10% experience BCR.^1^ The current guidelines recommend salvage brachytherapy as a good option for patients with LR after radical radiotherapy.^2^ Two types of salvage brachytherapy, including HDR and LDR, can be applied, with comparable cancer control.^3^ Regarding the PTV, although whole‐gland salvage brachytherapy is commonly performed for LR, focal salvage brachytherapy has been gradually used to reduce radiation‐related complications.^4^ However, the treatment strategies for LR that is refractory to focal salvage brachytherapy remain unclear.

In this report, we described a successful case of locally recurrent PCa treated with re‐salvage fLDR brachytherapy after salvage fLDR brachytherapy.

## Case presentation

**Fig. 1:**
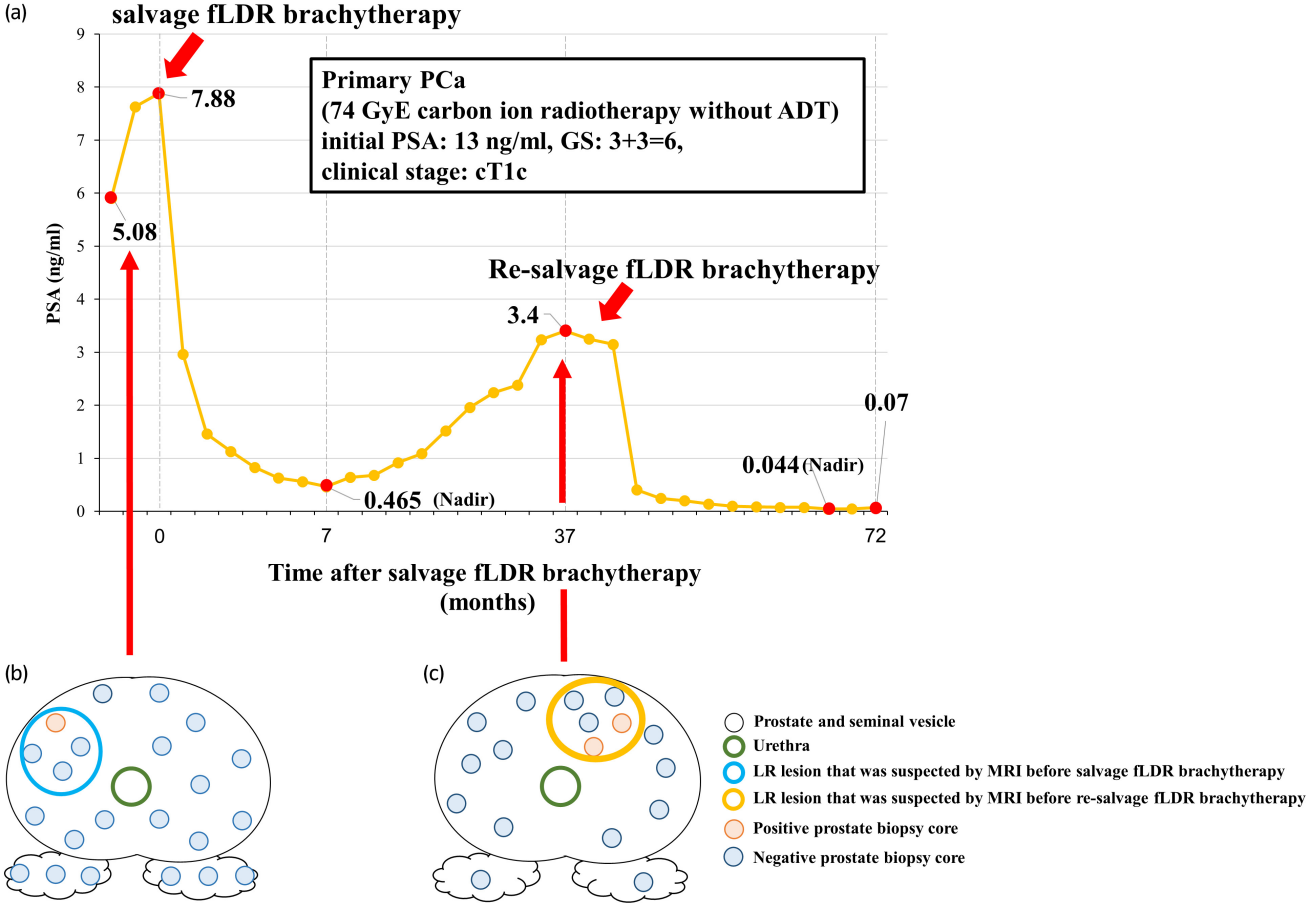
Changes in PSA after salvage fLDR brachytherapy and the locations of the positive/negative biopsy cores. (a) The patient was diagnosed with BCR, with a PSA level of 5.08 ng/mL, and was performed re‐salvage fLDR brachytherapy, with a PSA level of 7.88 ng/mL. At 7 months after salvage fLDR brachytherapy, the patient's PSA level decreased to 0.465 ng/mL (nadir), before gradually increasing to 3.4 ng/mL thereafter. At the 20‐month follow‐up after re‐salvage fLDR brachytherapy, the patient's PSA level decreased to 0.044 ng/mL and has remained at low levels. (b) The locations of the positive/negative biopsy cores before salvage fLDR brachytherapy. Of the 22 biopsy cores, one core with a suspected LR lesion on MRI revealed PCa. (c) The locations of the positive/negative biopsy cores before re‐salvage fLDR brachytherapy. Of the 17 biopsy cores, two cores with a suspected LR lesion on MRI revealed PCa. The cores that were biopsied within the target lesion of the salvage fLDR brachytherapy did not reveal PCa.

**Fig. 2:**
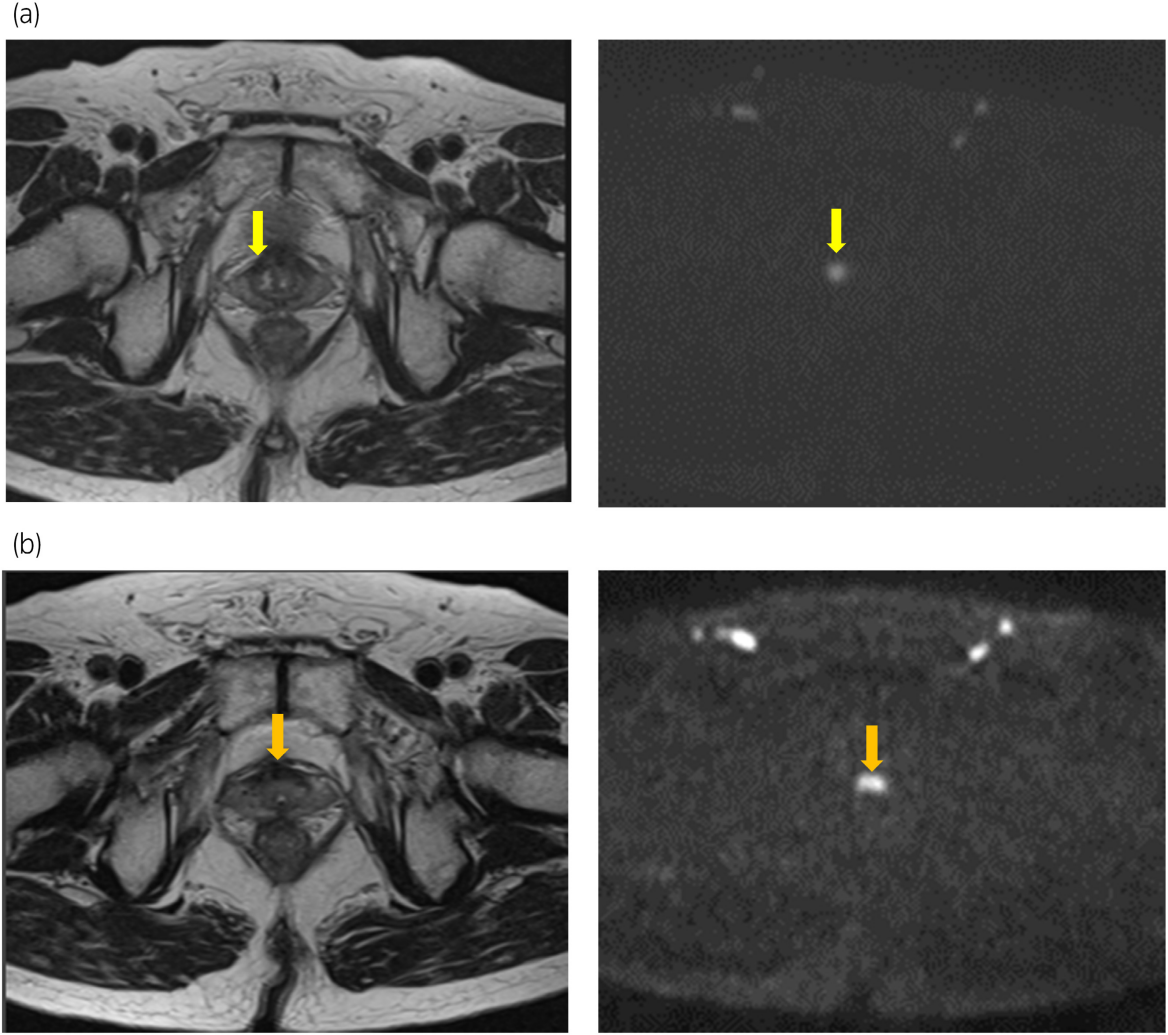
MRI images of LR. (a) MRI before salvage fLDR brachytherapy: LR (yellow arrow) was suspected on MRI because of the presence of a low signal intensity on T2‐weighted images and a high signal intensity on diffusion‐weighted imaging. (b) MRI before re‐salvage fLDR brachytherapy: LR (orange arrow), distant from the site treated with fLDR brachytherapy, was suspected on MRI.

A 77‐year‐old man, who had received carbon ion radiotherapy for localized PCa 12 years prior (Fig. 1a), was diagnosed with BCR (as defined by the Phoenix criteria). Although no distant recurrence was identified by CT and bone scintigraphy, LR was suspected by MRI (Fig. 2a). Subsequently, we performed a combination MRI‐targeted and systematic prostate biopsy, which revealed PCa with a GS of 4 + 4 (Fig. 1b). Consequently, the patient was administered salvage fLDR brachytherapy for the LR.

**Fig. 3:**
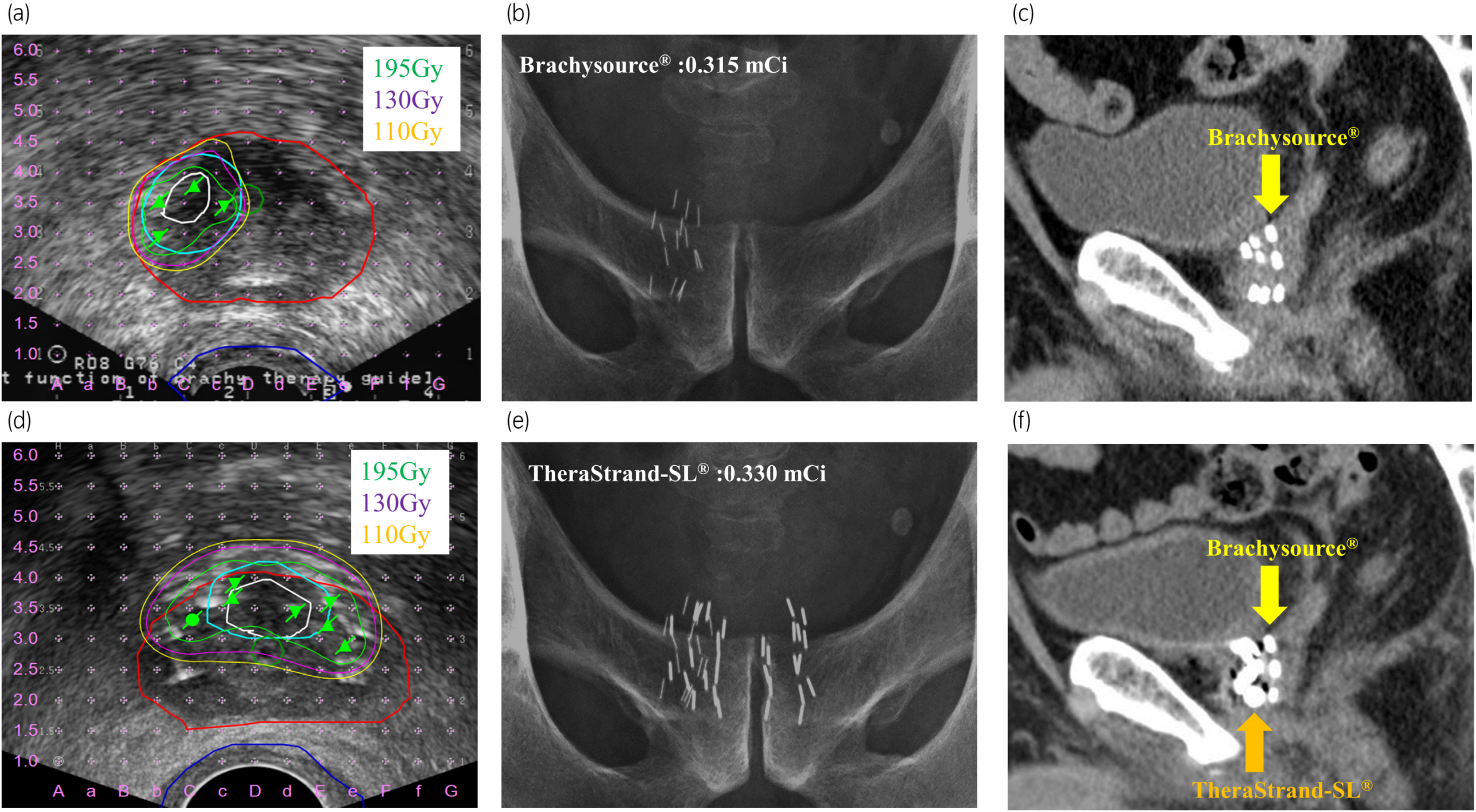
Dose distributions and pelvic X‐rays of the seed implantations. The red, green, blue, light blue, and white lines indicate prostate, urethra, rectum, PTV, and tumor based on MRI, respectively. (a) Intra‐implant dosimetry of salvage fLDR brachytherapy on transrectal ultrasound. The PTV was defined as the tumor area plus a 3‐mm margin on the anterior, posterior, right, and left sides based on multiparametric 3 Tesla MRI. (b) Pelvic X‐ray of the 15 seeds (Brachysource®) that were implanted in salvage fLDR brachytherapy. (c) Pelvic CT of the seeds (Brachysource®) that were implanted in the salvage fLDR brachytherapy. Brachysource® was displayed as an elongated high‐density area on the CT. (d) Intra‐implant dosimetry of re‐salvage fLDR brachytherapy on transrectal ultrasound. The PTV included the tumor site plus a 3‐mm margin on the anterior, right, and left sides based on multiparametric 3 Tesla MRI, but excluded the posterior margin to reduce the radiation dose to the urethra. (e) Pelvic X‐ray of the 30 seeds (TheraStrand‐SL®) that were implanted in re‐salvage fLDR brachytherapy, in addition to the 15 seeds implanted in salvage fLDR brachytherapy. (f) Pelvic CT of the seeds (TheraStrand‐SL®) that were implanted in the re‐salvage fLDR brachytherapy, in addition to other types of seeds that were implanted in the salvage fLDR brachytherapy. TheraStrand‐SL® was displayed as a thicker and rounder high‐density area than Brachysource® on the CT.

We performed salvage fLDR brachytherapy using the VariSeed® system and a real‐time ultrasound‐guided technique (Fig. 3a). We focally implanted 15 iodine‐125 seeds (Brachysource®), referring to ultrasound images fused with MRI images; the distribution of seeds on X‐ray after implantation and the dosimetric parameters are shown in Figure 3b and Table 1, respectively.

**Table 1:** Dosimetric parameters at the end of fLDR brachytherapy

	Salvage fLDR brachytherapy	Re‐salvage fLDR brachytherapy
PTV		
*D*_90_ (Gy)	121	146
*V*_150_ (%)	56.5	65
*V*_100_ (%)	87.3	96
Urethra		
UD_30_ (Gy)	77.5	117.2
Rectum		
*R*_100_ (cc)	0	0

After salvage fLDR brachytherapy, the PSA level declined briefly but then increased to BCR level (Fig. 1a). We next performed imaging examinations, which revealed a suspected recurrent lesion located at a different site from the previous recurrence without distant recurrence (Fig. 2b). The combination prostate biopsy was repeated, finding PCa with a GS of 4 + 4 (Fig. 1c). The patient was recommended to receive the relapse, but he refused due to the risk of sexual dysfunction. Therefore, we proposed re‐salvage fLDR brachytherapy, with a risk of radiation‐related toxicities, as an alternative, to which he consented.

We performed re‐salvage fLDR brachytherapy for the LR (Fig. 3d). We implanted 30 iodine‐125 seeds (TheraStrand‐SL®), which were different from those used in the previous salvage fLDR brachytherapy (Fig. 3e; Table 1).

After re‐salvage fLDR brachytherapy, the PSA level significantly decreased and has remained at low levels (Fig. 1a). The patient experienced urinary urgency and painful urination. These symptoms recovered within 6 months. Additionally, no GI toxicity was observed, and erectile function was preserved.

## Discussion

Although some studies on salvage brachytherapy for LR after radical radiotherapy have been reported,^3^ only a few have reported on re‐salvage brachytherapy for LR after salvage brachytherapy.^5^, ^6^ Maenhout *et al*.^5^ reported four cases who underwent re‐salvage focal HDR brachytherapy after salvage fLDR brachytherapy, resulting in two of the four cases experiencing no BCR. Moreover, Son *et al*.^6^ reported a case who received re‐salvage focal HDR brachytherapy for a LR after salvage focal HDR brachytherapy; at the 6‐month follow‐up, the patient had no BCR and no severe GU/GI toxicities. Thus, re‐salvage focal brachytherapy after salvage brachytherapy has acceptable oncological outcomes, suggesting that it has potential as a treatment strategy.

Both of these reports applied focal‐gland salvage brachytherapy rather than whole‐gland salvage brachytherapy due to a lower rate of GU/GI toxicities.^5^, ^6^ In whole‐gland salvage brachytherapy, grade 3 GI/GU toxicities have been reported in 15.7% and 2.8% of cases, respectively.^7^ However, literature on salvage fLDR brachytherapy after permanent LDR brachytherapy has reported no patients with grade 3 or more GI/GU toxicities.^8^ Indeed, in the present case, we only experienced one well‐controlled GU with no GI toxicities. Compared with whole‐grand salvage brachytherapy, the focal‐gland procedure can accurately adjust the radiation dose, which directly affects the urethra and rectum. Moreover, we excluded a posterior margin from the PTV to reduce the dose for the urethra at the re‐salvage brachytherapy, which may prevent high‐grade radiation‐related toxicity.

Imaging diagnosis of LR is almost always performed after diagnosis of BCR. However, Jansen *et al*.^9^ reported that PSMA PET scans of patients with no BCR after radical radiotherapy for PCa revealed metastasis in 50.8%. This report suggests that the indication for salvage brachytherapy cannot be determined based on BCR. Michael *et al*.^10^ reported that if PSADT is less than 6 months after radiotherapy, there is a high possibility of metastasis. In this case, the PSADT immediately before re‐salvage fLDR brachytherapy was 10 months, which was more than 6 months, and no BCR occurred after re‐salvage fLDR brachytherapy. PSADT might be a determining factor in the indication for salvage brachytherapy.

The specific definition of the target volume range of focal‐gland brachytherapy has not yet been established. The consensus meeting has proposed the following three categories of the target range: (i) ultra‐focal therapy: the tumor site and margin are targeted using MRI, (ii) focal therapy: half the gland is targeted, and (iii) focused therapy: the tumor site identified by MRI is targeted to receive the full dose, while the remaining gland receives a lower dose.^11^ In this regard, ultra‐focal therapy was selected for both LR in our case. Yamada *et al*.^12^ reported the oncological outcomes of PCa patients who were treated with the three types of salvage fLDR brachytherapy, in which the indications were determined according to the GS and positive core distribution in the prostate (i.e., ultra‐focal, hemi‐focal, and focused). However, further studies are needed to determine the appropriate methodology of the target volume range in focal‐gland salvage brachytherapy.

Another advantage of salvage fLDR brachytherapy is the ability to avoid initiating ADT for LR. According to a previous report, 93.5% of patients selected ADT for BCR after primary radiation therapy.^13^ However, ADT causes a significant reduction in a patient's sexual quality of life due to the induction of hypotestosteronemia.^14^ For patients, similar to our case, who desire to preserve male sexual function, (re‐)salvage fLDR brachytherapy may represent an effective alternative to ADT for the treatment of LR.

Regarding the unique aspect of our re‐salvage fLDR brachytherapy, we changed the type of seed from that used in the previous salvage fLDR brachytherapy. The type of seeds can be visually identified not only by X‐ray but also by CT (Fig 3c,f). Accordingly, we could manually correct the seeds in the post‐planning by referring to the location of the seeds on the CT/X‐ray, even in areas that overlapped with both seeds, providing an accurate assessment of the actual dose delivered to the prostate and surrounding structures.

## Conclusion

Re‐salvage fLDR brachytherapy represents a feasible option for LR after salvage fLDR brachytherapy. This treatment may avoid radiation‐related complications and the initiation of ADT, which, together with having favorable oncological outcomes, preserves sexual function.

## Author contributions

Takahiro Nakamoto: Conceptualization; writing – original draft. Takashi Yoshida: Conceptualization; writing – review and editing. Toshiko Shiga: Writing – review and editing. Makoto Taguchi: Writing – review and editing. Takao Mishima: Writing – review and editing. Shigenari Kawakita: Writing – review and editing. Takashi Murota: Supervision; writing – review and editing. Hidefumi Kinoshita: Supervision; writing – review and editing.

## Conflict of interest

The authors declare no conflict of interest.

## Approval of the research protocol by an Institutional Reviewer Board

The study was approved by the Institutional Review Board of Kansai Medical University General Medical Center (approval number: 2022253).

## Informed consent

Not applicable.

## Registry and the Registration No. of the study/trial

Not applicable.
